# Labelling people as ‘High Risk’: A tyranny of eminence?

**DOI:** 10.1136/bjsports-2015-095286

**Published:** 2015-11-16

**Authors:** Teppo L N Järvinen

**Keywords:** Bone, Cardiovascular, Cholesterol, Economics, Effectiveness

Whenever a doctor cannot do good, he must be kept from doing harm.

Hippocrates

Nowadays, being at ‘high risk’ of having a disease has become a disease in and of itself. Sweeping educational programmes at all levels of healthcare now turn an otherwise healthy person's ‘high’ blood pressure, elevated serum lipids or low bone density into chronic conditions having increased risk of a potentially bad event.^1^ But what represents ‘high risk’? This question lies at the heart of modern medicine, particularly with respect to pharmacological primary prevention.

Advocates of this evolution argue simply that primary prevention saves lives. However, permissive labelling of conditions as diseases may not be entirely harmless. On an individual patient level, possible disadvantages include making relatively healthy individuals perceive themselves as ‘sick’, and almost every treatment has inherent risks.^2^ On a societal level, we probably all still remember the discouraging effect of the new European guidelines on cardiovascular disease classified most adult Norwegians—among the healthiest populations in the world—to be at ‘high risk’ of cardiovascular disease.^3^ If these guidelines were applied to the Norwegian healthcare system, the focus on hypertension would drain the entire primary healthcare budget.

## ‘High risk': How low can we go?

Current debate on ‘High Risk as a Disease’ twirls around the ‘appropriate’ threshold for defining something as a disease. The recently introduced osteoporosis guideline put out by medical experts of the National Osteoporosis Foundation (NOF, USA) recommends osteoporosis medications if a person's 10-year probability of sustaining a hip fracture is 3% or over. Applying these NOF recommendations to a large prospective cohort study, at least 72% of Caucasian women >65 years of age and 93% of those >75 years of age, in the USA, would be recommended for drug therapy.^4^ The new cholesterol guideline similarly colonises virtually the entire elderly population into the realm of ‘sick’.

## Understanding risk: are the blind leading the blind?

But who are the right people to determine the threshold for ‘high risk’? Advocates of the hegemony of medical experts argue that doctors—as content experts—should define diseases.^5^ If we assume doctors are truly more competent in making value judgements about the lives of their patients than the patients sitting in front of them, should we not have proof that doctors can do the job? Sadly, despite medical education and clinical experience, doctors do not seem to possess the required skill.^6^

Even more discouragingly, patients might not fully agree with our perceptions. For example, the above noted NOF threshold is more than 15 times *lower* than what patients would consider a 10-year fracture risk justifying initiation of bone-targeted pharmacotherapy (50%).^7^

Similarly, there is a huge gap on what the two stakeholders consider ‘effective treatment’. Patients generally expect >20% *absolute* risk reduction (in heart attacks) for preventive pharmacotherapy to opt for treatment.^8^ In contrast, doctors world-wide began prescribing enthusiastically when an osteoporosis drug was shown to increase the probability of avoiding a hip fracture from 97.9% to 98.9%. Indeed, this 1% *absolute* risk advantage convinced our peers; mind you, after being framed as a 50% *relative* risk reduction.

## Does the Hippocratic Oath oblige us to intervene?

One may wonder why we still intervene when not only our patients disagree with our views on what constitutes a plausible risk to be treated or an effective treatment but also when the preventive efforts are 10 times more resource consuming than the treatment for the event to be prevented? Most doctors argue that, in matters of life and death, we have no other option. However, in other disciplines affecting the health and well-being of humans, we easily make decisions based on cost. For example, teachers in primary school are well aware of the fact that there are numerous children in each class with learning disabilities who might come from troubled families and who would be naturally at ‘high risk’ of becoming illiterate. But does the increased risk of consequent social deprivation drive us to fund and execute large-scale special needs teaching for all ‘at-risk kids’?

## Treating ‘high risk’—a mockery of shared decision-making?

**Figure 1:**
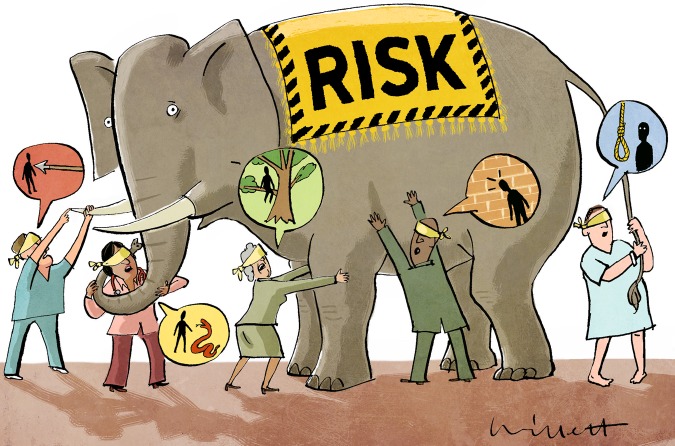
Risk is the probability that something bad or unpleasant will happen. Intuitively, it seems like a very simple concept. However, there is strong evidence that both doctors' and patients' comprehension of RISK is poor.

Let us get back to the pivotal question: Is treating ‘High Risk’ a viable concept? A strategy of obtaining a single estimate of a patient's risk and reducing this risk effectively is appealing. However, evidence from behavioural sciences suggests that we are generally poor in making probability decisions.^9^ And, despite laudable efforts to improve the communication and comprehension of both the concept of risk and the anticipated treatment benefit, risk-illiteracy of the gravest magnitude still affects both doctors and patients (figure 1).^6^ But without accurate and *common* comprehension of these key aspects, there is no basis for shared decisions.^10^ And without shared decision-making, pharmacological primary prevention becomes a tyranny of eminence.
